# Back beliefs in patients with low back pain: a primary care cohort study

**DOI:** 10.1186/s12891-019-2925-1

**Published:** 2019-12-01

**Authors:** Søren Grøn, Rikke Krüger Jensen, Tue Secher Jensen, Alice Kongsted

**Affiliations:** 1 Nordic Institute of Chiropractic and Clinical Biomechanics, Syddansk Universitet, Campusvej 55, 5230 Odense M, Denmark; 2 Department of Sports Science and Clinical Biomechanics, University of Southern Denmark, Odense, Denmark; 3 Department of Diagnostic Imaging, Silkeborg Regional Hospital, Silkeborg, Denmark; 4 Department of Clinical Medicine, Aarhus University, Aarhus, Denmark

**Keywords:** Back beliefs, Back pain, Primary care

## Abstract

**Background:**

The Back Belief Questionnaire (BBQ) measures beliefs about negative consequences of back pain. The aim of this study was to describe the back beliefs of a large clinical population with low back pain (LBP), to investigate the associations between back beliefs and patient characteristics when care-seeking, and between on-going pain and back beliefs at follow up.

**Methods:**

Patients aged over 18, consulting with LBP with or without radicular pain of all symptom durations, were recruited from chiropractic clinics. The BBQ was completed on the first visit and at 3- and 12-month follow-ups. Sociodemographic- and symptom-related questions were answered at baseline. A BBQ sum score was calculated at all three time points, and linear regression was used to analyse the cross-sectional association between baseline patient characteristics and BBQ scores. Wilcoxon signed-rank test was used to test differences in BBQ scores for patients with and without on-going LBP at 3- and 12-months follow up.

**Results:**

The baseline population consisted of 2295 participants. The median BBQ sum scores at baseline, 3 and 12 months had interquartile ranges of 33 [29–36], 33 [29–37], and 31 [27–35] respectively. Patient characteristics and symptoms were associated with baseline BBQ scores (*p* < 0.05), but most association were weak. The strongest association was with severe disability (4.0 points (95% CI 3.3–4.6) lower BBQ than no disability). Negative beliefs were related to more severe LBP at baseline and with on-going pain at follow up.

**Conclusion:**

At a population level, back beliefs were generally positive and relatively constant over time, but misconceptions about a poor prognosis were common. Studies exploring individual patterns of back beliefs and associations with clinical outcomes over time are recommended.

## Background

Low back pain (LBP) is the number one cause of disability worldwide and almost everyone will, at some point in their life, experience LBP [1]. LBP often follows an episodic pattern with most episodes being benign [2–4]. However, some episodes do become persistent and some people develop disability along with their LBP.

One important factor that can affect the development of disability in people with LBP is their beliefs about their back pain [5, 6]. One way of measuring these beliefs is via the Back Belief Questionnaire (BBQ), developed in 1996 [7]. It is a questionnaire consisting of statements regarding perceived inevitable negative consequences of an episode of back pain, such as “*Back trouble means periods of pain for the rest of one’s life*” or “*Once you have had back trouble there is always a weakness*” [7]. The BBQ has been widely used and has previously been validated showing acceptable psychometric properties considering unidimensionality, internal consistency and reliability [7–9].

Negative back beliefs as measured by the BBQ are associated with history of pain, care seeking behaviour and poorer outcomes from LBP such as increased levels of disability and pain [7, 8, 10–13]. Similarly, maladaptive illness perceptions are associated with higher levels of pain and lower physical function in patients with musculoskeletal pain [14]. Furthermore, low levels of pain self-efficacy defined as “*the beliefs held by people with chronic pain that they can carry out certain activities, even when experiencing pain*” is identified as a link between pain and disability [5], which is likely to be influenced by beliefs about one’s health condition.

Although, the evidence suggests that negative back beliefs are related to LBP and disability, there is still a lack of high-quality studies exploring back beliefs in clinical populations. A recent review found that the general population seems to agree largely with beliefs that back pain has inevitable negative consequences [6]; however, these data were mostly based on cohorts recruited more than a decade ago (1997–2010). Patients with persistent LBP have been shown to perceive of their back pain in a similar way to a ‘broken machine’, which was attributed to what they had learned from health care professionals [15]. For clinicians not to impose negative beliefs and to address those that are present, there is a need to understand how beliefs are developed and what type and extent of negative beliefs may be detrimental to recovery in people seeking care for LBP.

Therefore, the aim of this study was to describe back beliefs in a mixed clinical LBP population of chiropractic patients and investigate if specific patient characteristics were associated with negative beliefs. The specific objectives were to 1) describe back beliefs as measured by the BBQ when patients initially seek care, and at 3 months and 12 months after initiating care, 2) to investigate to what extent negative back beliefs were associated with a patient’s baseline characteristics and symptoms, and 3) to investigate if BBQ at follow up differed between people who had recovered from pain and those still reporting pain.

## Methods

The study was an observational cohort study reported according to the overall recommendations from ‘STrengthening the Reporting of OBservational studies in Epidemiology’ (STROBE) [16].

### Setting

This study was conducted using a subsample of data from the Danish Chiropractic low back pain Cohort (ChiCo) collected from November 1st 2016 to September 6th 2018, which was prior to the completion of enrolments in ChiCo. ChiCo is a longitudinal observational cohort of chiropractic patients recruited from 10 private chiropractic clinics in the Central Denmark Region. Patients comprising the cohort responded to questionnaires at their initial visit for an LBP episode and after 2 weeks (unrelated to this study), at 3 months and 12 months. They received treatment at the discretion of the chiropractor. Treatment was not affected by study participation. A Danish version of the BBQ was incorporated into the ChiCo at baseline, at 3-month and 12-month follow ups. Data collection was performed electronically using REDCap licensed by the Odense Patient Explorative Network (OPEN) [17]. ChiCo data are stored and managed at the Nordic Institute of Chiropractic and Clinical Biomechanics (NIKKB) with the University of Southern Denmark (SDU) as the responsible data authority (Danish Data Protection Agency, j.nr.: 2015-57-0008/16–47,215).

### Participants

To be eligible for inclusion, patients needed to be above the age of 18 and to consult the chiropractic clinic with a new episode of LBP, including both non-specific LBP and LBP with radicular pain. In this context we defined a new episode as initiating treatment for LBP and patients already in a course of treatment were not eligible. Patients were not included if LBP was suspected to be caused by serious pathology or immediate referral for surgery was required, this would also mean exclusion if occurring after study participation had started. Furthermore, the patients needed to understand and read Danish and have access to email.

### Data collection

The receptionist screened patients for inclusion criteria, informed them about the study and invited those eligible to participate. The baseline survey was divided into two parts. The first part involved the patient filling out the questionnaire on a tablet in the waiting room just before their initial consultation with the chiropractor. Written information about study participation and rights of participants was provided at the beginning of the survey and additional information was provided by the chiropractor during the consultation. On the day of the consultation, participants received a link to the second part of the baseline survey and a link to follow-up surveys was mailed after 3 months and 12 months. Within a few days after enrolment in the study, a research assistant called the participants to welcome them to the study, answer practical questions about participation, repeat the rights related to study participation and remind them to fill out the second part of the baseline survey if they had not already done so.

### Variables

#### Back belief questionnaire

The translation of the BBQ was conducted as recommended by forward and back translation [18]. The forward translation was performed by two persons with Danish as their first: A back pain researcher who is familiar with English as working language and a layperson who has a master’s degree in English Literature. After the independent translations of the questionnaire, the translations were compared, and a common version agreed on. The back translation was performed by two persons who are native English speakers and have lived in Denmark and used Danish for more than 10 years. One is a back-pain researcher and one is a layperson. The wording of the back translated version was compared to the original BBQ and two of the authors (AK and TSJ) decided on the pre-final version. The pre-final version was tested among a group of 10 people, five with content knowledge and 5 people without. Based on comments from this group, the final version was decided on by AK and TSJ. The internal consistency of the scale was tested in the study sample (see “Statistical Analyses”). Additional validation of the translated BBQ has not been performed. The original BBQ has previously been validated by the developers who found it to be one-dimensional and showing acceptable internal consistency and reliability [7]. Similar results were found in a validation study of the BBQ providing evidence supporting the structure of the BBQ [8].

The BBQ was answered in the first part of the baseline survey and again after 3 and 12 months. It consists of 14 statements regarding perceived inevitable negative consequences of an episode of LBP with five of these acting as distractors. Each statement is rated on a five-point Likert scale scored from 1 (completely disagree) to 5 (completely agree). Scores are then reversed and summed up to a final score ranging from 9 to 45 with lower scores indicating more negative beliefs about back pain.

To our knowledge, there is no consensus on the cut-point for negative versus positive back beliefs. In this study, we chose a sum score above 27 to indicate positive beliefs, as this cut-point was used in a recent systematic review on back beliefs [6]. When assessing individual items, we interpreted a score of 1 or 2 (on a reversed scale) as agreeing with the statement, as this method has been used previously [13].

#### Additional baseline variables

From the first part of the baseline survey, the following measures were used: age and sex (from the patient’s personal identification (social security) number); duration of current pain episode (1–2 days, 3–7 days, 1–2 weeks, 2–4 weeks, 1–3 months, 3–12 months, more than a year); number of days with pain last year (≤30 days, > 30 days); back pain intensity (Numerical Rating Scale (NRS) asking about ‘typical back pain’ the previous week. 0 = No pain; 10 = Worst imaginable pain); disability measured by the 23-item Danish Roland-Morris Disability Questionnaire (RMDQ, scale 0–23) [19]. The NRS and the RMDQ have been used widely in previous research and have been validated [19–21]. Previous episodes of LBP (none, 1 episode, 2–3 episodes, more than 3 episodes); whether or not the patient had attended other care for their current LBP episode (none, general practitioner, physiotherapist, another chiropractor, other) and whether or not the patient had previously attended a clinician for LBP (no care-seeking, general practitioner, physiotherapist, chiropractor, other clinician) were from the second part of the baseline survey.

BBQ was filled out in the first part of the questionnaire before the patient’s initial consultation with the chiropractor in order to prevent potential impact on back beliefs resulting from the contact. The additional baseline variables were chosen because they were considered likely to influence back beliefs which we have seen in previous studies using similar variables [7, 8, 10–13].

### Study sample

The sample size was decided based on other purposes of the cohort. To be able to detect a small to moderate effect size (Cohen’s f = 0.10) in the present study using linear regression (F-test) with a power of 0.90 at the 0.05 level of significance, a sample size of *n* = 146 was required for categorical variables with four levels [22]. Still, we used the full cohort to obtain as precise estimates as possible. The minimum of observations at any time was *n* = 1613 (the regression analysis testing the association between BBQ and more than 3 episodes of LBP) and the smallest observed category in any analysis was *n* = 143 (other treatment for current LBP provided by a chiropractor, Table 3) .

### Statistical methods

Baseline characteristics were presented as means with standard deviations (SD) or proportions. Since enrolment for the ChiCo cohort was still ongoing, not all participants had reached 3- and 12-month follow ups, and follow-up rates at 3 and 12 months were calculated from the number of participants who had received the follow-up questionnaires. We used a Wilcoxon signed-rank test to test the difference in baseline BBQ sum scores between follow-up study populations and non-responders.

To confirm that the BBQ represented one latent variable, we calculated Cronbach’s alpha. All nine items of the BBQ had an alpha score above 0.7 and a total alpha score of 0.75, indicating acceptable internal consistency.

The BBQ was analysed in separate analyses for each of the three time points (baseline, 3 months, 12 months). First, we dropped observations with six or less answers of the BBQ items. For the remaining observations, we used chained multiple imputation to fill out missing items of the BBQ. The chained multiple imputation was based on the 14 BBQ items, back pain intensity and number of days with pain last year. Because the number of imputations was low, we extracted one dataset out of five imputed versions for the analyses instead of conducting analyses across multiple datasets.

Data on the BBQ was not normal distributed and back beliefs were presented at each time point as median sum scores with interquartile range (IQR), histograms of the BBQ sum score and as means for the individual items. We also calculated the percentage of participants who agreed with each item at each time point. To investigate the association between back beliefs and patients’ baseline characteristics and symptoms, we used univariate linear regression with the BBQ sum score at baseline as the dependent variable and other baseline characteristics as explanatory variables. Pain and disability were categorised by dividing the scores into quantiles and age was divided into three categories based on the inspection of a LOWESS plot (Locally Weighted Scatterplot Smoothing). Categorical explanatory variables were introduced as indicator variables using the lowest category as the reference (for example each of the 2nd to 4th quartile of pain scores were compared to the 1st quartile). Results were presented as regression coefficients with 95% confidence intervals and *p*-values. The linear relationship between pain intensity on a continuous scale and the BBQ were checked in a LOWESS plot. Testing the assumption of homoscedasticity, the Breusch-Pagan test that did not make us reject the null-hypothesis of equal variance (*p* = 0.53).

At the 3-month and 12-month follow ups, BBQ sum scores were described separately for people who reported LBP and for those who did not. Reporting LBP was defined as scoring 1 or higher on the NRS at the respective time points. The statistical significance of group differences was tested using the Wilcoxon signed-rank test.

All analyses were performed using Stata/MP 15.1 (StataCorp LLC, TX 77845, USA).

## Results

### Participants

A total of 2370 participants were available in the cohort at baseline. After excluding those responding to less than six BBQ items, 2293 (97%) participants were included in this study. Those excluded had similar characteristics to those included (Table 1). The participants included at baseline had a mean age of 44 years and 59% were men (Table 1). The second part of the baseline survey was answered by 1664 participants and of those the questions concerning previous treatment and receiving other treatment for previous LBP were answered by 1657 and 1659, respectively.

**Table 1:** Baseline Characteristics

Variable	Study sample at baseline (*n* = 2293)	Excluded (*n* = 77)
Age in years, mean (SD)	44 (14)	55 (15)
Age range in years	18–87	20–80
Females	41%	52%
Episodes of LBP before current one,		
None	17%	19%
1 episode	14%	6%
2–3 episodes	24%	25%
More than 3 episodes	45%	50%
Missing (n)	680	45
Time since start of current episode of LBP,		
1–14 days	60%	55%
2–4 weeks	11%	18%
1–12 months	18%	17%
More than a year	10%	11%
Missing (n)	14	4
Days with LBP during the previous year		
More than 30 days	37%	49%
Missing (n)	0	40
Back pain (VAS 0–10), mean (SD)	6.7 (2.1)	6.9
Back pain divided in quantiles		
0–5	26%	
6–7	34%	
8	23%	
9–10	17%	
Disability (RMDQ 0–23), mean (SD)	12.7 (5.5)	–
RMDQ score divided into quantiles		
0–9	29%	
10–14	28%	
15–17	20%	
18–23	23%	

*SD* standard deviation

*LBP* low back pain

*VAS* visual analogue scale

*RMDQ* Roland-Morris Disability Questionnaire

**Fig. 1:**
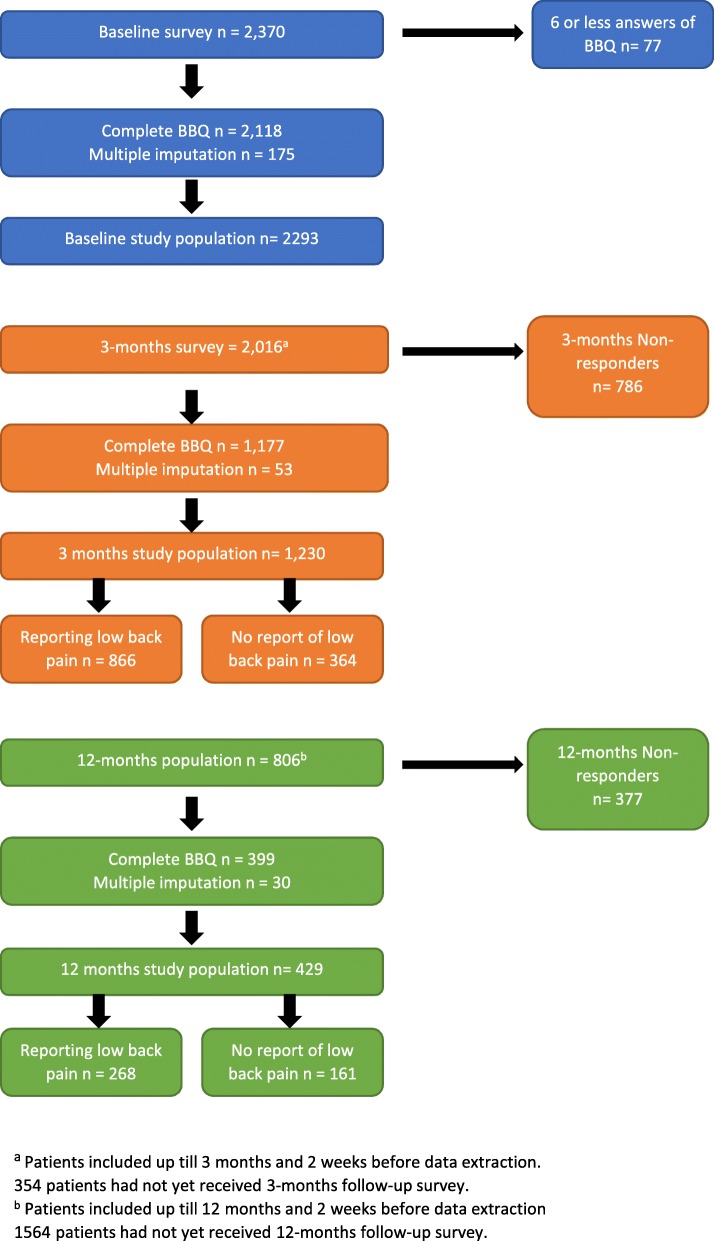
Study Flowchart

The study population at the 3-month follow up included 1230 participants (61% response rate) and at the 12-month follow up, 429 (53%) (Fig. 1). When comparing baseline characteristics of 3- and 12-month follow-up non-responders with the 3- and 12-month study population, there were no substantial differences (Appendix).

### Outcome data (Back beliefs)

**Fig. 2:**
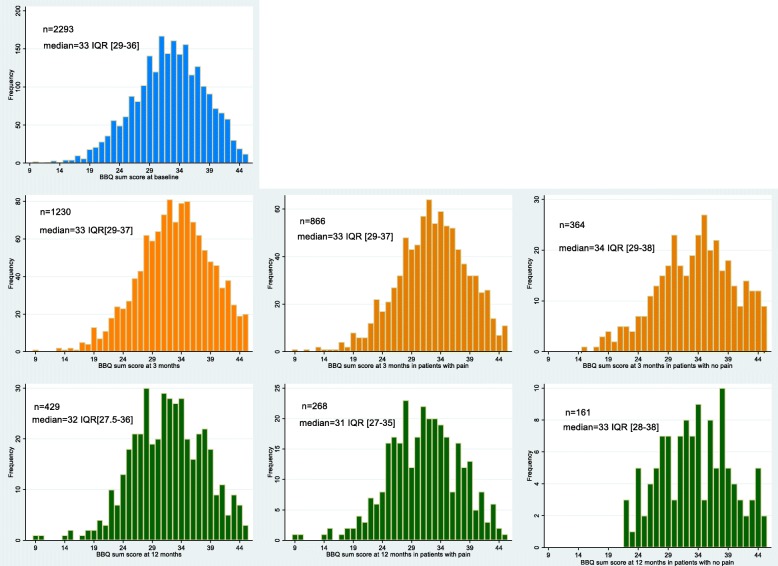
Histograms of the distribution of sum scores of the Back Belief Questionnaire

The median BBQ sum score was 33 IQR [29–36] at baseline, 33 IQR [29–37] at the 3-month follow up and 32 IQR [28–36] at the 12-month follow up. In general, the BBQ sum scores were high, indicating positive back beliefs, but with a wide span of observed scores (Fig. 2).

At all three time points, item 14 “*Later in life, back trouble gets progressively worse*” was the item that most patients agreed with (34, 26 and 31% agreeing at baseline, 3 months and 12 months, respectively), followed by item 6 “*Back trouble makes everything in life worse”* (32, 26 and 31% respectively) (Table 2).

**Table 2:** Scores on individual items of the Back Belief Questionnaire (BBQ)

			3 months						12 months					
ITEM	Baseline *n* = 2293		Total (n) = 1230		LBP, *n* = 866		no pain, *n* = 364		Total (n) = 429		LBP, *n* = 268		no pain, *n* = 161	
	mean	agree %	mean	agree %	mean	agree %	mean	agree %	mean	Agree %	mean	agree %	mean	agree %
BBQ 1 There is no real treatment for back trouble	4.3	6	4.3	8	4.1	9	4.5	5	4.2	9	4.0	10	4.5	7
BBQ 2 Back trouble will eventually stop you from working	3.8	19	3.8	17	3.8	16	3.9	18	3.5	26	3,5	25.	3.5	14
BBQ 3 Back trouble means periods of pain for the rest of one’s life	3.7	17	3.8	16	3.6	18	4.1	10	3.6	20	3.3	28.	4.2	6
BBQ4 Doctors cannot do anything for back trouble^a^	3.8	17	3.7	17	3.6	19	4.0	12	3.6	20	3.4	25	3.9	12
BBQ5 A bad back should be exercised^ab^	4.5	4	4.4	8	4.4	6	4.5	7	4.5	5	4.5	6	4.6	4
BBQ 6 Back trouble makes everything in life worse	3,1	32	3.3	26	3,3	24	3.2	32	3.1	31	3.1	31	3.1	32
BBQ 7 Surgery is the most effective way to treat back trouble^a^	4.4	3	4.3	4	4.3	8	4.3	4	4.2	5	4.2	6	4.3	3
BBQ 8 Back trouble may mean you will end up in a wheelchair	3.7	17	3.6	20	3,6	18	3.4	26	3.4	21	3.5	19	3.3	24
BBQ 9 Alternative treatments are the answer to back trouble^a^	3.7	18	3.3	16	3.3	16	3.3	15	3.3	17	3.3	16	3.2	17
BBQ 10 Back trouble means long periods of time off work	3,7	12	3.9	8	3.9	7	3.7	12	3.9	10	3.9	9	3.8	12
BBQ 11 Medication is the only way of relieving back trouble^a^	4.3	5	4.2	6.	4.2	6	4.4	4	4.2	6	4.2	6	4.2	6
BBQ 12 Once you have had back trouble there is always a weakness	3.7	17	3.6	18	3.5	20	3.9	13	3.5	21	3.4	24	3.7	17
BBQ 13 back trouble must be rested	3.3	23	3.4	20	3.4	20	3.5	20	3.4	19	3.4	20	3.6	18
BBQ 14 Later in life back trouble gets progressively worse	2.9	34	3.2	26	3.1	30	3.4	18	3.0	31	2.9	38	3.3	21

Mean: mean reversed score of each item of the BBQ with a high score indicating disagreement with the statement (range 1–5)

% agree: Percentage of the population agreeing with the statement (score of 1 or 2 indicates agreement)

^a^ distractor item and not part of the sum score

^b^BBQ5 is not reversed due to the context of the question, it is therefore also % of disagreement noted

Item 1 *“There is no real treatment for back trouble*” was the one that the fewest patients agreed with at all time points (6, 8 and 9% agreeing at baseline, 3 months and 12 months, respectively). It was followed by item 10 “*Back trouble means long periods of time off work*” (12, 8 and 10% respectively) (Table 2).

The 3- and 12-month study populations had slightly higher baseline BBQ scores as compared with those of the non-responders at 3 and 12 months. The 3-month study population had a median baseline BBQ sum score of 33 IQR [29–37] as compared with that of non-responders, 32 IQR {28–36] (*p* < 0.001). The median baseline BBQ sum score for the 12-month study sample was 33 IQR [29–37] and 31 IQR [27–36] for the 12-month non-responders (*p* < 0.001).

### Association between back beliefs and baseline characteristics

Baseline BBQ scores were associated with almost all the investigated patient characteristics with *p*-values < 0.05, but most associations were weak and explained little of the variance in BBQ-scores (Adj. R^2^ ranging from < 0.01 to 0.06) (Table 3). The strongest association was between negative beliefs and severe disability (− 4.0 (95% CI -4.6; − 3.3)) as compared with no disability, and also, a long history of LBP and high pain intensity were associated with negative beliefs. Female patients had more positive beliefs than males.

**Table 3:** Associations between baseline scores on the Back Belief Questionnaire and baseline characteristics. Univariate linear regressions with the BBQ sum score as dependent variable and baseline characteristics as explanatory variables

	Coefficient	P > t	95% Confidence Interval	Degrees of Freedom	Adjusted R-squared
Age				2	0.0087
18–37					
38–51	1.31	< 0.001	0,72; 1.89		
52–87	1.10	< 0.001	0.51; 1.70		
Sex (female)	1.46	< 0.001	0.97; 1.95	1	0.0143
More than 3 episodes of LBP	−1.29	< 0.001	− 1.85; − 0.72	1	0.0116
Pain for more than 1 year	−1.26	0.002	−2.06; − 0.46	1	0.0038
More than 30 days of pain	− 1.17	< 0.001	− 1.67; − 0.67	1	0.0087
Backpain (VAS)					
Backpain linear relation	−0.34	< 0.001	− 0.46; − 0.23	1	0.0140
Quantiles of back pain		< 0.001			
VAS 0–5				3	0.0133
VAS 6–7	−0.66		−1.29; 0.03		
VAS 8	−1.31		−2.0; −0.62		
VAS 9–10	− 2.1		− 2.85; −1.34		
Disability (RMDQ)					
Quantiles of RMDQ		< 0.001		3	0.0660
RMDQ score 0–9					
RMDQ score 10–14	−0.55		−1.17; 0.07		
RMDQ score 15–17	−2.03		−2.71; − 1.35		
RMDQ score 18–23	−3.96		−4.62; −3.31		
Other treatment for current LBP (*n* = 547) ^a^	−0.95	0.002	−1.54; − 0.36	1	0.0053
General practitioner (*n* = 308)	−1.59	< 0.001	−2.31; −0.88	1	0.0108
Chiropractor (n = 143)	−0.85	0.095	−1.85; 0.15	1	0.0011
Physiotherapist (*n* = 315	−1.05	0.004	−1.77; −0.34	1	0.0044
Other (*n* = 160)	−0.39	0.416	−1.34; 0.55	1	−0.0002
Having seen more than 1 other healthcare provider (*n* = 297)	−1.39	< 0.001	−2.12; −0.66	1	0.0078
Treatment for previous LBP (*n* = 1104 of 1657) ^b^	−0.53	0.082	−1.12; −0.07	1	0.0012
General practitioner (*n* = 322)	−1.60	< 0.001	−2.30; −0.90	1	0.0113
Chiropractor (*n* = 811)	−0.31	0.272	−0.87; 0.25	1	0.0001
Physiotherapist (*n* = 570)	−0.89	0.003	−1.47; −0.29	1	0.0046
Other (*n* = 207)	−1.01	0.019	−1.86; −0.17	1	0.0027
Having seen more than 1 other health care provider (*n* = 571)	−1.164	< 0.001	−1.75; −0.58	1	0.0085

*LBP* low back pain

*VAS* visual analogue scale

*RMDQ* Roland-Morris Disability Questionnaire

^a^out of a total of 1659 answers with a total of 634 missing

^b^out of a total of 1657 answers with a total of 636 missing

**Table 4:** Baseline characteristics by each follow-up population

Variable	Baseline study population *n* = 2293	3-month non-responders *n* = 786	3-month study population *N* = 1230	12-month non-responders *N* = 377	12-month study population *N* = 429
Age in years, mean (SD)	44(14)	40(13)	47(13)	41(14)	46(13)
Age range in years	18–87	18–80	18–80	18–80	18–80
Females	41%	38%	43%	41%	43%
Episodes of LBP before current one,					
None	17%	19%	16%	23%	20%
1 episode	14%	13%	14%	19%	12%
2–3 episodes	24%	25%	24%	20%	24%
> 3 episodes	45%	43%	46%	39%	45%
Total (n)	1613	344	1067	203	379
Time since start of current episode of LBP,					
1–14 days	60%	61%	61%	61%	61%
2–4 weeks	11%	10%	11%	9%	10%
1–12 months	18%	19%	18%	19%	20%
> a year	10%	11%	10%	11%	9%
Total (n)	2279	778	1221	375	425
Days with LBP during the previous year					
Less than or equal to 30 days	63%	60%	65%	63%	64%
> 30 days	37%	40%	35%	37%	36%
Total (n)	2293	350	1230	210	375
Back pain (VAS 0–10), mean	6.7	6.7	6.7	6.8	6.6
Disability (RMDQ 0–23), mean	12.7	11.9	12.8	12.2	12.3

*SD* standard deviation

*LBP* low back pain

*VAS* visual analogue scale

*RMDQ* Roland-Morris Disability Questionnaire

Prior to inclusion, 67% of the participants had sought care for previous LBP episodes and 33% had sought care from other health care providers for their current episode. No systematic relationship was observed between any previous care seeking and BBQ-scores, but more negative beliefs were reported by those who had seen a general practitioner as compared to those who had not consulted general practice (Table 3). Those who had seen another health care provider for their current episode of LBP scored 0.95 lower on BBQ (95% CI -1.54; − 0.36) as compared to those who had not, with consulting a general practitioner having the strongest negative association with beliefs (Table 3). Patients who had visited more than one kind of health care provider for their current or a previous episode of LBP had lower scores on the BBQ compared with those who had consulted none or only one health care provider (Table 3).

### Back beliefs at follow-up differentiated between patients with or without LBP

At the 3-month follow up, 866 (70%) patients reported LBP and at the 12-month follow up, 268 (62%) reported LBP (Fig. 1). At 3 months, those who did not report LBP had a median BBQ sum score of 34 IQR [29–38] and those who did report LBP had a median BBQ sum score of 33 IQR [29–37] (*p* < 0.01). At 12 months, the median BBQ sum score was 33 IQR [28–38] for the group reporting no LBP and 31 IQR [27–35] for the group reporting LBP (*p* < 0.01).

At both 3-and 12-month follow ups, participants without LBP agreed mostly with item 6 “*Back trouble makes everything in life worse”* (32% at both 3 and 12 months) followed by item *8 “Back trouble means you will end up in a wheelchair”* (26 and 24% respectively)*.* At 3 months, the fewest number of participants agreed with item 1 (5%) followed by item 3 “*Back trouble means periods of pain for the rest of one’s life*” (10%). At 12 months, the fewest number of participants agreed with item 3 (6%) followed by item 1 (7%) (Table 2).

The participants still reporting LBP at follow up agreed overall with the same individual items as the baseline study cohort (Table 2).

## Discussion

### Key results

This is the first study to explore back beliefs in a clinical cohort of chiropractic patients at different time points during and after care-seeking. With reference to the objectives of this study we found that the participants largely disagreed with the inevitability of negative consequences of back pain and, at a population level, BBQ scores did not differ greatly between baseline and 3- or 12-month follow ups. Patients with high pain intensity and high disability had the most negative back beliefs. Interestingly, we found back beliefs to only differ very little between those who had recovered and those who still reported pain at follow up.

### Limitations and generalizability

The original BBQ has been validated but in this study we used a translated version of the BBQ that has not been validated. This is a limitation that might affect the results, and these should therefore be interpreted with caution. However, the translation process was done using forward and backwards translation, which have previously been used to translate the BBQ into French with good results [9] and we therefore relied on the original validation. The uncertainty of generalizability to chiropractic patients in general should also be noted since data were from a limited number of clinics. Still, we have no reasons to believe that these were not representative of Danish chiropractic clinics. We do not consider the results necessarily generalizable to patient populations from other settings**.** A study comparing chiropractic patients with patients of general practice, found a clear difference between the two populations and thus illustrates that despite similar complaints, research results are not directly transferable between different practice types [23].

### Interpretation

The item most often agreed with was “*Later in life back trouble gets progressively worse*”. Although back pain may become somewhat more disabling with age [24, 25], this is an overly negative expectation, since most episodes of back pain have a very good prognosis and back pain often follows a fluctuating trajectory for many years which can get gradually worse [26]. Hence, there seems to be a need for educating people more about the prognosis of back pain.

Even participants who did not report pain at follow up often agreed with “*Back trouble makes everything in life worse*” indicating that although they had recovered, they still remembered their episode of LBP with all its negative aspects. Somewhat surprisingly, one in four of those who had recovered from pain at the follow ups agreed with the statement “*Back trouble means you will end up in a wheelchair*” which may indicate that some patients had developed unnecessary fear about LBP despite recovering from it previously. This again stresses the importance of clinicians explaining back pain as a non-progressive disease.

Bostick et al. found that people with LBP who sought medical care had more positive beliefs compared with those who did not [8]. It might be that they had received useful information regarding their pain, or perhaps care seekers in general are more resourceful or seek care because of a positive expectation to benefit from treatment. In our study, all the participants were seeking care and we saw more negative back beliefs among participants who had previously visited more than one kind of health care provider. These patients might belong to a subgroup of more complex LBP for whom some of the negative beliefs reflect their actual experience, and where multiple health care providers have not been able to change that perception.

Our findings suggest that, in general, chiropractic patients in Denmark seem to have more positive beliefs about back pain than those found amongst the general population in a systematic review by Morton et al. [6]. This review found a mean BBQ sum score below 27 in eight out of 12 studies concluding that people from the general population on average have negative back beliefs. However, most of the studies included in the review were based on older cohorts (included 1997 to 2010) and therefore were not directly comparable with our cohort. In recent years, there has been a shift towards promoting positive messages about back pain, self-management of LBP and moving away from the ‘broken machine’-model [15]. This may be what we now see reflected as more positive beliefs. Another possible explanation is that chiropractic patients comprise a subpopulation of LBP patients with high expectations about effective care and sufficient resources to pay for treatment. Chiropractic patients are overall more educated and have less severe pain than patients presenting to general practice [23].

Back beliefs differed very little between those who had recovered and those who still reported pain at follow up, with the observed difference being due to very negative expectations only being present in those still reporting pain. Positive beliefs in people who have recovered from LBP have previously been observed [17–19], and point to the importance of a positive personal experience. As this study occurred at a population level, we do not know if people who did not recover and reported negative beliefs at follow up held on to such beliefs or shifted towards them. This should be explored by investigating individual trajectory patterns.

Previous studies have found associations between negative back beliefs and both high disability or high pain intensity [7, 10–13]. In our study, we found the strongest association between high disability and negative back beliefs. There is a close link between pain and disability, and it is not surprising that these are associated with lower BBQ scores. How these aspects of LBP are linked should be investigated in future studies exploring the longitudinal relationships between pain, disability and beliefs.

A strength of this study was the size of the population. A smaller sample was available at follow up, but the samples were sufficiently large to describe back beliefs at the investigated time points, and those included at follow up did not differ substantially from the baseline population that was not followed. We therefore decided that the sample was adequate for the study before follow up had been completed. We also consider it a strength that the participants answered the BBQ before their first consultation with the chiropractor, and thus, the baseline BBQ scores were not influenced by the beliefs of the chiropractor.

## Conclusion

This study demonstrated generally positive back beliefs in patients seeking chiropractic care, and at a population level, similar beliefs at the initial visit and at 3 and 12 months after seeking care. Those with negative beliefs were patients with disabling LBP, a long history of LBP, and many previous health care visits. Future studies should investigate the course of back beliefs in individual patients and explore the longitudinal relationship between developing back beliefs and clinical outcomes. Importantly, clinicians should be aware that many patients have overly negative expectations about the prognosis of LBP. Further investigation is required to explore to what extent the negative beliefs related to health care visits might be a consequence of these contacts.

## Data Availability

The datasets used and/or analysed during the current study are available from the corresponding author on reasonable request.

## Abbreviations

BBQ: Back Belief Questionnaire
ChiCo: Danish Chiropractic low back pain Cohort
CI: Confidence interval
IQR: Interquartile range
LBP: Low back pain
LOWESS: Locally Weighted Scatterplot Smoothing
NIKKB: Nordic Institute of Chiropractic and Clinical Biomechanics
NRS: Numerical Rating Scale
OPEN: The Odense Patient Explorative Network
RMDQ: Roland-Morris Disability Questionnaire
SD: Standard deviation
SDU: University of Southern Denmark

## References

[1] GBD 2015 Disease and Injury Incidence and Prevalence Collaborators (2016). Global, regional, and national incidence, prevalence, and years lived with disability for 310 diseases and injuries, 1990–2015: a systematic analysis for the Global Burden of Disease Study 2015. Lancet (London, England).

[2] Kongsted A, Hestbaek L, Kent P (2017). How can latent trajectories of back pain be translated into defined subgroups?. BMC Musculoskelet Disord.

[3] Henschke N, Maher CG, Refshauge KM, Herbert RD, Cumming RG, Bleasel J (2008). Prognosis in patients with recent onset low back pain in Australian primary care: inception cohort study. BMJ (Clinical research ed).

[4] Maher C, Underwood M, Buchbinder R (2017). Non-specific low back pain. Lancet (London, England).

[5] Lee H, Hubscher M, Moseley GL, Kamper SJ, Traeger AC, Mansell G (2015). How does pain lead to disability? A systematic review and meta-analysis of mediation studies in people with back and neck pain. Pain.

[6] Morton L., Bruin M., Krajewska M., Whibley D., Macfarlane G.J. (2018). Beliefs about back pain and pain management behaviours, and their associations in the general population: A systematic review. European Journal of Pain.

[7] Symonds TL, Burton AK, Tillotson KM, Main CJ (1996). Do attitudes and beliefs influence work loss due to low back trouble?. Occup Med (Oxford, England).

[8] Bostick GP, Schopflocher D, Gross DP (2013). Validity evidence for the back beliefs questionnaire in the general population. Eur J Pain (London, England).

[9] Dupeyron A, Lanhers C, Bastide S, Alonso S, Toulotte M, Jourdan C (2017). The Back belief questionnaire is efficient to assess false beliefs and related fear in low back pain populations: a transcultural adaptation and validation study. PLoS One.

[10] Ng SK, Cicuttini FM, Wang Y, Wluka AE, Fitzgibbon B, Urquhart DM (2017). Negative beliefs about low back pain are associated with persistent high intensity low back pain. Psychol Health Med.

[11] Elfering A, Mannion AF, Jacobshagen N, Tamcan O, Muller U (2009). Beliefs about back pain predict the recovery rate over 52 consecutive weeks. Scand J Work Environ Health.

[12] Elfering A, Muller U, Rolli Salathe C, Tamcan O, Mannion AF (2015). Pessimistic back beliefs and lack of exercise: a longitudinal risk study in relation to shoulder, neck, and back pain. Psychol Health Med.

[13] Urquhart DM, Bell RJ, Cicuttini FM, Cui J, Forbes A, Davis SR (2008). Negative beliefs about low back pain are associated with high pain intensity and high level disability in community-based women. BMC Musculoskelet Disord.

[14] de Raaij EJ, Ostelo RW, Maissan F, Mollema J, Wittink H (2018). The Association of Illness Perception and Prognosis for pain and physical function in patients with noncancer musculoskeletal pain: a systematic literature review. J Orthop Sports Phys Ther.

[15] Setchell J, Costa N, Ferreira M, Makovey J, Nielsen M, Hodges PW (2017). Individuals' explanations for their persistent or recurrent low back pain: a cross-sectional survey. BMC Musculoskelet Disord.

[16] von Elm E, Altman DG, Egger M, Pocock SJ, Gotzsche PC, Vandenbroucke JP (2008). The Strengthening the reporting of observational studies in epidemiology (STROBE) statement: guidelines for reporting observational studies. J Clin Epidemiol.

[17] Department of Clinical Research UoSD, Odense. Odense Patient Data Explorative Network (OPEN). Available from: https://open.rsyd.dk/. Accesed 3 Sept 2019.

[18] Beaton DE, Bombardier C, Guillemin F, Ferraz MB (2000). Guidelines for the process of cross-cultural adaptation of self-report measures. Spine.

[19] Albert HB, Jensen AM, Dahl D, Rasmussen MN (2003). Criteria validation of the Roland Morris questionnaire. A Danish translation of the international scale for the assessment of functional level in patients with low back pain and sciatica. Ugeskr Laeger.

[20] Williamson A, Hoggart B (2005). Pain: a review of three commonly used pain rating scales. J Clin Nurs.

[21] Childs JD, Piva SR, Fritz JM (2005). Responsiveness of the numeric pain rating scale in patients with low back pain. Spine.

[22] Faul F, Erdfelder E, Buchner A, Lang AG (2009). Statistical power analyses using G*power 3.1: tests for correlation and regression analyses. Behav Res Methods.

[23] Hestbaek L, Munck A, Hartvigsen L, Jarbol DE, Sondergaard J, Kongsted A (2014). Low back pain in primary care: a description of 1250 patients with low back pain in danish general and chiropractic practice. Int J Family Med.

[24] Wong AY, Karppinen J, Samartzis D (2017). Low back pain in older adults: risk factors, management options and future directions. Scoliosis Spinal Disord.

[25] Leboeuf-Yde C, Fejer R, Nielsen J, Kyvik KO, Hartvigsen J (2011). Consequences of spinal pain: do age and gender matter? A Danish cross-sectional population-based study of 34,902 individuals 20-71 years of age. BMC Musculoskelet Disord.

[26] Kongsted A, Kent P, Axen I, Downie AS, Dunn KM (2016). What have we learned from ten years of trajectory research in low back pain?. BMC Musculoskelet Disord.

